# Clinical characteristics of outpatients and inpatients with COVID-19 in Bushehr: a report from the south of Iran

**DOI:** 10.2217/fvl-2020-0231

**Published:** 2021-01-25

**Authors:** Mohsen Keshavarz, Ahmad Tavakoli, Sareh Zanganeh, Mohammad Javad Mousavi, Katayoun Vahdat, Mehdi Mahmudpour, Iraj Nabipour, Amirhossein Darabi, Saeid Keshmiri

**Affiliations:** 1 ^1^The Persian Gulf Tropical Medicine Research Center, The Persian Gulf Biomedical Sciences Research Institute, Bushehr University of Medical Sciences, Bushehr, Iran; 2 ^2^Research Center of Pediatric Infectious Diseases, Institute of Immunology & Infectious Diseases, Iran University of Medical Sciences, Tehran, Iran; 3 ^3^Department of Medical Virology, Faculty of Medicine, Iran University of Medical Sciences, Tehran, Iran; 4 ^4^Bacteriology & Virology Department, Shiraz Medical School, Shiraz University of Medical Sciences, Shiraz, Iran; 5 ^5^Department of Immunology & Allergy, Faculty of Medicine, Bushehr University of Medical Sciences, Bushehr, Iran; 6 ^6^Faculty of Medicine, Bushehr University of Medical Sciences, Bushehr, Iran

**Keywords:** clinical features, computed tomography, COVID-19, hospitalization, Iran, laboratory markers, SARS-CoV-2, severe acute respiratory syndrome coronavirus 2

## Abstract

**Aim:** To investigate clinical, laboratory and imaging features of COVID-19 patients in Bushehr, a southern province of Iran. **Materials & methods:** A total of 148 COVID-19 patients were enrolled. The patients were categorized into four groups including inpatients, outpatients, elderly and nonelderly. Clinical, laboratory and computed tomography characteristics were analyzed and compared. **Results:** Levels of erythrocyte sedimentation rate, CRP, lactate dehydrogenase and aspartate aminotransferas among inpatients were higher than outpatients. There were significant differences in the levels of creatinine and blood urine nitrogen between elderly and nonelderly patients. The incidence of ground-glass opacities in inpatients was significantly higher than in outpatients. **Conclusion:** COVID-19 is associated with more severe renal failure in elderly patients. Elderly patients with underlying conditions are at increased risk of severe progression of COVID-19.

In December 2019, a series of fatal pneumonia cases of unknown etiology was identified in Wuhan City, Hubei Province of China. In January 2020, the Chinese authorities reported that the causative agent was a previously unknown coronavirus, severe acute respiratory syndrome coronavirus 2 (SARS-CoV-2). In February 2020, the WHO officially named the disease caused by the SARS-CoV-2 as coronavirus disease-2019 (COVID-19) [1,2]. Up to 11 October 2020, COVID-19 had been recognized in 216 countries and territories, with a total number of reported laboratory-confirmed cases being nearly 37 million and deaths being more than one million [3]. Iran is the most affected country by COVID-19 in the Eastern Mediterranean region and the first cases of the disease were reported on 20 February 2020. According to the last report of the WHO, published on 12 October 2020, there were 496,253 laboratory-confirmed cases of the SARS-CoV-2 and 28,293 deaths due to the COVID-19 infection in Iran [3].

Based on the genome sequence data, it has been revealed that SARS-CoV-2 is a member of the genus *Betacoronavirus* within the family *Coronaviridae* [4,5]. It is an enveloped virus with a single strand, positive-sense RNA genome. Following transmission by inhalation of respiratory droplets, contact with contaminated surfaces or fecal-oral route, and other means, viral replication takes place in cells of the respiratory and GI tracts [5]. The average incubation period of the disease is reported to be 2–14 days, however, there is evidence that it can last as long as 19–27 days [6]. The clinical manifestations of COVID-19 can range from asymptomatic mild disease to severe respiratory failure requiring hospital admission and mechanical ventilation [7]. The main clinical symptoms in COVID-19 patients are fever, cough, fatigue and sore throat [8]. Significant differences in the clinical and demographic characteristics of COVID-19 patients have been observed in different parts of the world.

Up till now a few studies have reported clinical characteristics and laboratory data of COVID-19 in Iran. Shahriarirad *et al.* have performed a multicenter retrospective study on 113 hospitalized COVID-19 patients in Fars province, located in the south of Iran [9]. In another study conducted by Nikpouraghdam *et al.*, data regarding the epidemiological features of 2968 COVID-19 patients in a hospital in Tehran (capital city and located in the central part of Iran) have been reported [10]. Javanian *et al.* has also performed a retrospective cohort study on the clinical symptoms and laboratory characteristics of 100 COVID-19 patients in Babol, in the North of Iran [11]. Although several studies have been reported, data on COVID-19 patients in different parts of Iran, no study has characterized features of COVID-19 patients in Bushehr, a southern province of Iran. This study aimed to investigate the clinical, laboratory and imaging findings of COVID-19 patients in Bushehr. The patients were categorized into groups including inpatient, outpatient, elderly and nonelderly, and results were compared between the four groups.

## Materials & methods

### Study design & population

From 29 April to 30 May 2020, a total of 900 patients suspected of COVID-19 infection were admitted to the Shohadaye-Khalije-Fars Hospital, Nabi Akram Health Center, and Shahid Ganji. These three designated hospitals are responsible for the management of COVID-19 patients in Bushehr province, Iran, assigned by the Iranian Ministry of Health. Patients were divided into two groups, inpatients and outpatient. In our study, patients presenting with mild or moderate symptoms, such as fever, cough, sore throat and dyspnea were categorized into the outpatient group, while patients with severe symptoms like SpO2 <94%, PaO2/FiO2 <300 mmHg and respiratory frequency >30 breaths per minute were inpatients. Furthermore, the patients were also categorized into two groups, elderly (≥60 years old) and nonelderly (<60 years old) to compare clinical, laboratory and computed tomography (CT) findings between different ages. Both oropharyngeal and nasopharyngeal specimens were collected for each patient and combined into a viral transport medium to be tested as a single sample for SARS-CoV-2. Samples were transferred to the virology department of Bushehr University of Medical Sciences under refrigeration. The study was approved by the Ethics Committee of Bushehr University of Medical Sciences (ethic code number: IR.BPUMS.REC.1399.004), and written informed consent was obtained from each participant before enrollment.

### Data collection

Patients were diagnosed with COVID-19 infection, according to the criterion of the WHO interim guidance [12]. For patients with confirmed COVID-19 infection, blood tests were performed on complete blood count analyzer (Sysmex Corporation, Kobe, Japan) and SELECTRA automated analyzer (VitaLab, Dieren, The Netherlands), and all data including laboratory results, epidemiological and demographic characteristics, clinical signs and symptoms, and underlying conditions were recorded. Routine blood examinations were erythrocyte sedimentation rate (ESR), complete blood count, serum biochemical tests (including blood urea nitrogen, Creatinine, Creatine Phosphokinase, lactate dehydrogenase (LDH), aspartate aminotransferase (AST), alanine transaminase, C-reactive protein).

### CT examinations

The chest CTs were taken from all suspected individuals. For all individuals, chest CT evaluation was done on a 16-row multidetector scanner (Siemens Sensation 16, Erlangen, Germany) with the following parameters: 120 kV, 100–150 mA, 0.6 mm collimation and 1:1 pitch. Two radiologists with more than 5 years of experience evaluated the CT findings in consensus and CT findings were classified as ground-glass opacities (GGOs), consolidation, consolidation with GGOs, pleural effusion, ground glass and pleural effusion and normal.

### Statistical analysis

Statistical analysis was performed using Statistical Package for the Social Sciences (SPSS) for Windows release 25.0 (SPSS Inc., IL, USA). Descriptive statistics (mean, standard deviation [SD], CI) were generated for demographic and clinical characteristics. The normal distribution of parameters was analyzed using the Kolmogorov–Smirnov test. Since the distribution of the variables did not follow a normal distribution, nonparametric tests were applied. Mann–Whitney U test was used to compare the measurements between groups. Odds ratio with a 95% CI was used to evaluate the effect and association of qualitative data. p-values < 0.05 were considered to indicate significant statistically.

## Results

### Demographic characteristics

From 29 April to 30 May 2020, a total of nine hundred cases suspected of COVID-19 infection were admitted to our hospitals. After screening, 148 patients (16.4%) were confirmed to have COVID-19 infection by real-time reverse transcription-PCR (rRT-PCR); 53 (35.8%) of those 148 patients were outpatients and 95 (64.2%) were inpatients. The ages of patients with COVID-19 infection ranged from 1 to 90 years (mean age of 44.49 ± 17.4 years), and over half of the patients (52.7%) were men. The mean age of the subjects in the inpatient and outpatient groups were 48.89 ± 17.69 and 38.00 ± 15.78, respectively (p < 0.001). According to the sex distribution, inpatients were composed of 48 (50.5%) males and 47 (49.5%) females. However, 30 (56.6%) males and 23 (43.4%) females were included in the outpatient group (Table 1). In the inpatient group, 5 (5.2%) death cases were seen, while no death case was detected in the outpatients.

**Table 1.** Association of the baseline data and clinical manifestations of the study subjects based on the type of admission (inpatient or outpatient) and age group.

Characteristic	Category	Type of admission			Age group		
		Inpatient (n = 95)	Outpatient (n = 53)	p-value	Nonelderly (<60) (n = 118)	Elderly (≥60) (n = 30)	p-value
Sex	Male	48 (50.5%)	30 (56.6%)	0.477	66 (55.9%)	12 (40%)	0.119
	Female	47 (49.5%)	23 (43.4%)		52 (44.1%)	18 (60%)	
Coexisting conditions	Hypertension	5 (5.2%)	0 (0%)	–	1 (0.8%)	4 (13.3%)	0.003
	Type 2 diabetes	7 (7.3%)	1 (1.9%)	0.122	5 (4.2%)	3 (10%)	0.066
	Chronic lung diseases	4 (4.2%)	0 (0%)	–	1 (0.8%)	3 (10%)	0.01
	Cardiovascular diseases	7 (7.3%)	0 (0%)	–	4 (3.4%)	3 (10%)	0.041
	Chronic blood disorders	1 (1%)	0 (0%)	–	1 (0.8%)	0 (0%)	–
	Hepatitis B	1 (1%)	0 (0%)	–	0 (0%)	1 (3.3%)	–
	Bladder cancer	1 (1%)	0 (0%)	–	0 (0%)	1 (3.3%)	–
	Down syndrome	1 (1%)	0 (0%)	–	1 (0.8%)	0 (0%)	–
	None	68 (71.5%)	52 (98.1%)	Reference^†^	105 (89%)	15 (50%)	Reference^†^
Symptoms	Fever	67 (70.5%)	27 (51%)	0.018	72 (61%)	22 (73.3%)	0.211
	Cough	62 (65.3%)	21 (39.6%)	0.003	63 (53.4%)	20 (66.7%)	0.191
	Dyspnea	54 (56.8%)	22 (41.5%)	0.075	60 (50.8%)	16 (53.3%)	0.808
	Sore throat	12 (12.6%)	10 (18.9%)	0.309	19 (16.1%)	3 (10%)	0.402
Radiological findings	GGO	33 (34.7%)	7 (13.2%)	0.0008	31 (26.3%)	9 (30%)	0.418
	Consolidation	4 (4.2%)	1 (1.9%)	0.215	4 (3.4%)	1 (3.3%)	0.837
	Consolidation plus GGO	14 (17.7%)	2 (3.8%)	0.012	12 (10.2%)	4 (13.3%)	0.41
	Pleural effusion	1 (1%)	0 (0%)	–	0 (0%)	1 (3.3%)	–
	GGO and pleural effusion	1 (1%)	0 (0%)	–	0 (0%)	1 (3.3%)	–
	Normal	42 (44.2%)	43 (81.2%)	Reference^†^	71 (60.2%)	14 (46.7%)	Reference^†^
Outcome	Alive	90 (94.8%)	53 (100%)	–	116 (98.3%)	27 (90%)	0.025
	Dead	5 (5.2%)	0 (0%)		2 (1.7%)	3 (10%)	

† The base state for statistical comparison.

GGO: Ground glass opacity.

### Comorbidities & clinical manifestations

Inpatients showed underlying medical conditions more frequently than the outpatients. The most common coexisting medical conditions in the inpatient group were Type 2 diabetes (n = 7; 7.3%) and cardiovascular diseases (n = 7; 7.3%), followed by hypertension (n = 5; 5.2%) and chronic lung diseases (n = 4; 4.2%). Among outpatients, only 1 (1.9%) patient had Type 2 diabetes, whereas 52 patients (98.1%) did not present underlying diseases (Table 1).

Fever was the most common clinical presentation among both inpatients and outpatients (70.5 and 51%, respectively). The cough was the second most frequently observed symptom among inpatients, found in 65.3% of patients, while it was observed in 39.6% of outpatients. Dyspnea was more common among inpatients (56.8%) than in outpatients (41.5%) (Table 1).

### Laboratory findings

Considering the blood leukocytes, the count of WBCs had no significant difference between the two groups (6.60 ± 3.31 vs 6.41 ± 2.12; p = 0.39). However, lymphocyte counts were lower in the inpatient group in comparison with the outpatient group (1.36 ± 0.76 vs 2.04 ± 0.90; p < 0.0001). Concerning the inflammatory markers, ESR was significantly higher in the inpatients in comparison with the outpatients (44.99 ± 26.48 vs 17.95 ± 17.97; p < 0.0001). Additionally, CRP level was significantly higher among the inpatients than the outpatients (49.02 ± 35.72 vs 15.49 ± 24.55; p < 0.0001; Table 2). Both blood urine nitrogen (BUN) and creatinine levels were higher in the inpatients in comparison with the outpatients, but the differences were not statistically significant (p = 0.84 and p = 0.48, respectively). Moreover, CPK levels did not have a significant change between the two groups (p = 0.41). However, the LDH level was higher among the inpatients than the outpatients (559.57 ± 380.54 vs 358.97 ± 119.83; p < 0.0001). Among the liver enzymes, the AST level was significantly higher in the inpatient group compared with the outpatient group (38.84 ± 38.77 vs 26.16 ± 13.66; p < 0.001). However, no statistically significant difference was detected between the two groups (36.77 ± 45.99 vs 30.16 ± 22.38; p = 0.39; Table 2).

**Table 2.** Baseline data and paraclinical findings of the study participants.

Characteristic	Type of admission			Age		
	Inpatient (n = 95)	Outpatient (n = 53)	p-value^†^	Nonelderly (n = 118)	Elderly (n = 30)	p-value^†^
Age (year)	48.89 ± 17.69	38.00 ± 15.78	<0.001	38.03 ± 12.6	70.15 ± 8.14	<0.0001
WBC (10^9^/l)	6.60 ± 3.31	6.41 ± 2.12	0.39	6.42 ± 2.84	6.94 ± 3.3	0.882
Lymphocyte (10^9^/l)	1.36 ± 0.76	2.04 ± 0.90	<0.0001	1.69 ± 0.92	1.27 ± 0.58	0.002
ESR (mm/h)	44.99 ± 26.48	17.95 ± 17.97	<0.0001	29.52 ± 23.98	56.65 ± 27.25	<0.0001
CRP (mg/l)	49.02 ± 35.72	15.49 ± 24.55	<0.0001	31.62 ± 34.22	57.09 ± 35.39	<0.001
BUN (mg/dl)	17.36 ± 13.26	14.09 ± 4.28	0.84	15.12 ± 10.96	20.1 ± 10.69	<0.001
Creatinine (mg/dl)	1.13 ± 0.67	0.98 ± 0.23	0.48	1.01 ± 0.45	1.3 ± 0.82	0.003
CPK (IU/l)	192.98 ± 244.41	138.35 ± 148.30	0.41	175.62 ± 229.36	166.84 ± 165.08	0.958
LDH (U/l)	559.57 ± 380.54	358.97 ± 119.83	<0.0001	480.01 ± 351.58	520.11 ± 226.51	0.049
AST (U/l)	38.84 ± 38.77	26.16 ± 13.66	<0.001	35.45 ± 36.25	30.5 ± 13.93	0.969
ALT (U/l)	36.77 ± 45.99	30.16 ± 22.38	0.39	36.67 ± 43.4	26.46 ± 16.83	0.146

† Nonparametric, Mann–Whitney U was used for between groups analysis.

ALT: Alanine Aminotransferase; AST: Aspartate aminotransferase; BUN: Blood urine nitrogen; CRP: C-reactive protein; CPK: Creatine kinase; ESR: Erythrocyte sedimentation rate; LDH: Lactate dehydrogenase; WBC: White blood cell.

Regarding the age group, results showed a significant increase in the levels of ESR among the elderly patients (56.65 ± 27.25 mm/h) compared with the nonelderly patients (29.52 ± 23.98 mm/h) (p < 0.0001). Also, the levels of CRP were significantly different between these groups, so that a higher level was observed among the elderly patients (57.09 ± 35.39 mg/l) than the nonelderly ones (31.62 ± 34.22 mg/l). Similarly, higher levels of BUN and LDH were seen in elderly patients compared with nonelderly patients (Table 2).

### Comparison of radiographic findings

**Figure 1.**
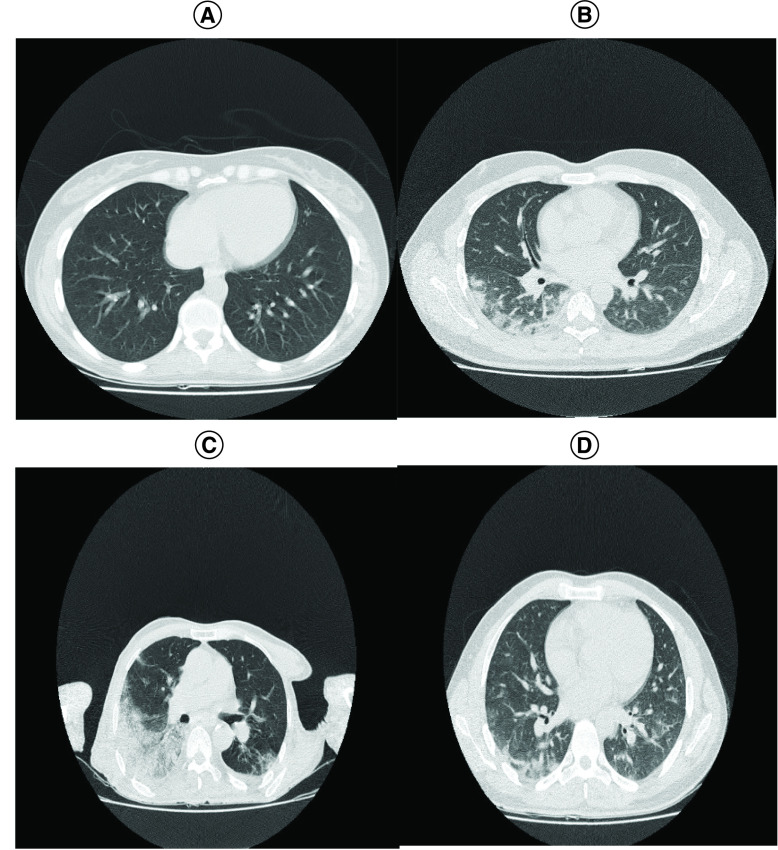
Representative computed tomography images of patients with coronavirus disease-2019. **(A)** Female, 27 years old (outpatient) with fever and dyspnea. Axial ccmputed tomography (CT) image showed normal lung markings. **(B)** Female, 35 years old (inpatient with alive outcome) with fever and dyspnea. Axial CT image showed small bilateral areas of ground-glass opacities. **(C)** Male, 74 years old (elderly) with fever, cough and dyspnea. Axial CT image shows larger ground-glass opacities in the right lung. **(D)** Male, 31 years old (nonelderly) with fever and cough. Axial CT image shows ground-glass opacities in the bilateral lungs.

According to the chest CT examinations, GGOs were observed in 33 (34.7%) inpatients and 7 (13.2%) outpatients and the difference was statistically significant (p = 0.0008). Additionally, consolidation in conjunction with ground glass opacities was detected in 14 (17.7%) inpatients and 2 (3.8%) outpatients and the difference was statistically significant (p = 0.012). Also, according to the comparison based on the age of patients, no significant findings were seen in lung CT scans (Table 1 & Figure 1).

## Discussion

In the face of the great threat posed by COVID-19 to human health, laboratory assessment and early prognosis of the patient’s condition should be given more attention. Currently, the rapid spread of COVID-19 in Bushehr, a province in southern Iran, has led to a dramatic increase in the number of hospitalized patients as well as increased mortality in recent weeks. In this descriptive study, 148 patients confirmed with COVID-19 were evaluated. Patients were divided into the outpatient and inpatient groups, and clinical symptoms, laboratory parameters and chest CT features were evaluated.

Since SARS-CoV-2 is a novel pathogen, pre-existing immunity is not present in the human community, which makes all humans susceptible to the infection. In high-risk groups such as elderly people and individuals with co-existing comorbidity, the outcome of infection with SARS-CoV-2 will be more serious [13]. Our results showed that among underlying medical comorbidities, Type 2 diabetes, cardiovascular diseases and hypertension were the most common in all patients with COVID-19. Besides, the frequency of comorbidities was higher in the inpatients compared with the outpatients. These results are consistent with the findings of another study conducted by Nikpouraghdam *et al.* in Iran [10]. They found that diabetes, hypertension and cardiovascular diseases are among the most common comorbidities in COVID-19 patients. In another investigation performed by Norooznezhad *et al.*, on 431 patients with a definite diagnosis for COVID-19 infection, underlying medical comorbidities such as cardiovascular diseases and diabetes were the most frequently identified comorbidities [14]. Shahriarirad *et al.* carried out a study on 113 hospitalized confirmed cases of COVID-19 admitted to hospitals in Shiraz [9]. Similar to our findings, hypertension, diabetes and cardiovascular diseases were the most prevalent underlying comorbidities among hospitalized patients with COVID-19.

Laboratory parameters are pivotal in helping us to identify COVID-19 cases and can use in the understanding of the disease course, prognosis and the decision-making process. As a prominent result, a significant decrease in lymphocyte counts among the inpatients compared with the outpatients were observed, and the decrease was more remarkable in elderly patients. In parallel with our findings, several recent studies have been suggested that lymphopenia is a strong indicator of infection with COVID-19 [15–18]. According to the results of a recent meta-analysis, COVID-19 patients presenting lymphopenia have an approximately fivefold increased risk to develop a severe form of the disease [19]. Several potential mechanisms leading to the lymphocyte depletion have been proposed such as direct infection and death of lymphocytes by the virus, destroying lymphatic organs and lymphocytes inhibition by metabolic molecules produced by metabolic disorders [20]. Tavakolpour *et al.* hypothesized several possible underlying causes for the lymphopenia in severe COVID-19 patients including the inflammatory cytokine storm, exhaustion of T cells, direct infection of T cells by SARS-CoV-2 and interference with T cell expansion [21].

Levels of ESR, CRP, LDH and AST among inpatients were higher than the outpatients in our study, and the results are in accordance with the previous studies. It has been documented that elevated LDH levels are associated with worse outcomes in COVID-19 patients [22]. LDH is a metabolic enzyme that is found in nearly all major organ cells and its elevation is usually indicative of tissue damage. The SARS-CoV-2 binds to angiotensin converting enzyme 2 (ACE2) receptor on human lung, and accordingly, the lung is the first affected organ. However, abnormalities in cytokine profiles and dysfunction in multiple organs can be found when the disease progresses [23], suggesting the virus can affect other organs. In addition to the lungs, ACE2 is expressed on other tissues such as heart, kidney and GI tract [24] and this can explain why multiple organs are afflicted during COVID-19 infection.

One of the striking findings of the present study is that there was no significant difference in the levels of creatinine and BUN between the inpatients and outpatients, while the difference was observed between elderly and nonelderly patients. Consistent with our results, a study conducted by Guo *et al.* showed a higher median level of creatine and BUN than young patients [25]. BUN is a well-known biomarker of kidney function, and these findings indicate that COVID-19 infection is associated with more severe renal failure and abnormalities in elderly patients than young ones.

Our findings showed that the prevalence of GGO and pulmonary consolidations were significantly higher among inpatients compared with outpatients. This is in line with the results of the recent meta-analysis conducted by Sun *et al.* They reported that GGO, consolidation, and GGO plus consolidation were the most common findings reported in 94.5% of the studies [26]. Also based on another comprehensive study conducted by Güneyli *et al.*, the most common CT findings of COVID-19 patients were GGO, consolidation and GGO plus consolidation [27].

There are several limitations to our study that should be considered. First, due to the study design, some important laboratory parameters and biomarkers were not measured in our patients, such as D-dimer, B-type natriuretic peptide (BNP), N-terminal pro b-type natriuretic peptide (NT-proBNP) and pocalcitonin. Second, because of the small sample size in the study, our analyses are low in power. Third, the number of patients under the age of 18 was limited, therefore, we could not perform any analysis on pediatric patients with COVID-19.

## Conclusion

Based on our data, underlying diseases were more frequent in inpatients than in outpatients. The most common underlying diseases in the inpatient group were Type 2 diabetes, cardiovascular diseases, hypertension and chronic lung diseases. Fever and cough were the most common symptom observed among inpatients. Moreover, the levels of BUN and creatinine in elderly patients were higher than in young patients, suggesting that COVID-19 infection is associated with more renal failure in elderly patients. Furthermore, most of the hospitalized patients had GGO or mixed GGO and consolidation lesions in the chest CT findings.

**Summary points**

- From 29 April to 30 May 2020, a total of 148 patients confirmed with coronavirus disease-2019 infection were admitted to three hospitals in Bushehr, a province in the south of Iran.
- Among the patients, 35.8% were outpatients and 64.2% were inpatients.
- The mean age of inpatients and outpatients was 48.89 ± 17.69 and 38.00 ± 15.78, respectively.
- The frequency of underlying disease among inpatients was higher than outpatients.
- The most common underlying disease in all patients was Type 2 diabetes.
- Ground glass opacities were observed in lung CT images of 34.7% of inpatients and 13.2% of outpatients.
- Levels of erythrocyte sedimentation rate, CRP, lactate dehydrogenase and aspartate aminotransferas among inpatients were higher than outpatients.
- A significant increase was seen in the levels of erythrocyte sedimentation rate, blood urine nitrogen, CRP and lactate dehydrogenase among elderly patients compared with nonelderly patients.
